# Supplementary material for: Capacitive NO2 Detection Using CVD Graphene-Based Device
Source: Nanomaterials (Basel). 2023 Jan 5;13(2):243. doi: 10.3390/nano13020243 (PMC9863439; doi:10.3390/nano13020243)
Supplement: Supplementary file 1 [file nanomaterials-13-00243-s001.zip › nanomaterials-2110408-supplementary.pdf]

nanomaterials

Supplementary Materials

Capacitive NO_2 Detection Using CVD Graphene-Based Device

Wonbin Ju [1] and Sungbae Lee [2,*]

[1] Department of Physics and Photon Science, Gwangju Institute of Science and Technology, Gwangju 61005, Republic of Korea
[2] Korea Institute of Energy Technology, KENTECH College, Naju 58330, Jeonnam, Republic of Korea
* Correspondence: jaylinlee@kentech.ac.kr

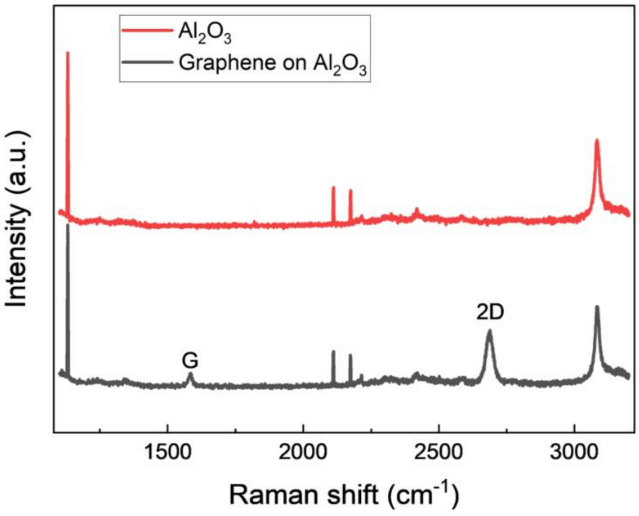

Figure S1. Raman spectroscopy of the Al_2O_3 substrate and the graphene on the substrate measured using 532 nm laser at 5 mW at a range of 1000–3000 cm^{-1}. The intensity ratio of 2D and G peaks is 4.5, indicating that the graphene samples used in our experiments are monolayer graphenes.

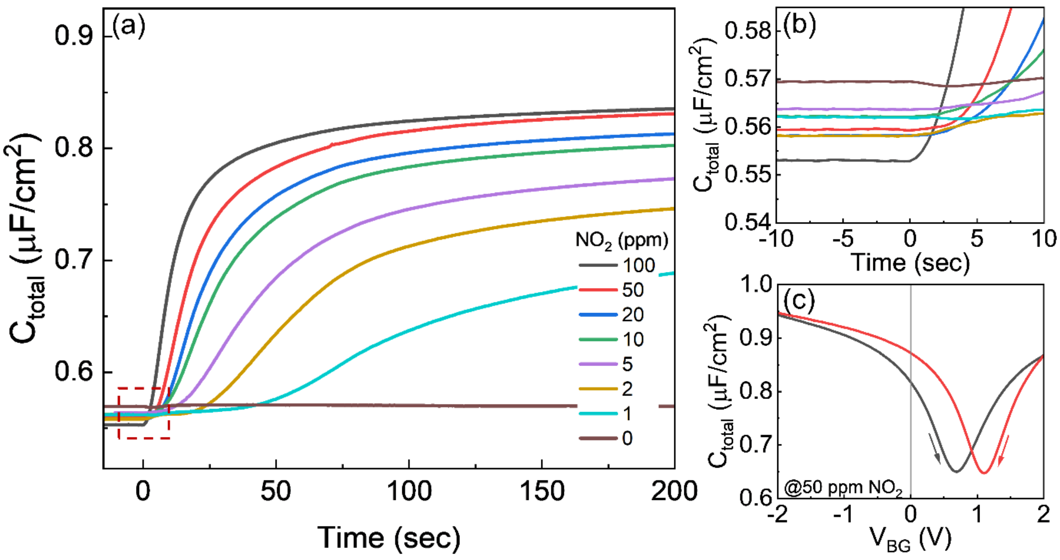

Figure S2. (**a**) Changes in C_{total} when the test chamber is filled with 0–100 ppm NO_2. The response plot in Figure 4a is from the one converted from Figure S2a using equation (1). All initial C_{total} before NO_2 exposure is between 0.55 and 0.57 μF/cm^2. All the initial values are not identical as shown in Figure S2b due to experimental reason. To make C_{total} before NO_2 exposure be identical, recovery optimization needs to be further studied. This is not covered in this work because the study on recovery optimization is out of the scope of this article. The C_{total} corresponding to the response values measured after 2 h exposure to NO_2 in Figure 6a is explained by gate-dependent C_{total} in

Figure 6b. Discrepancies between C_{total} measured after 2-hour exposure and C_{total} at V_{BG} = 0 V are observed in Figure 6b. The discrepancy comes from the hysteresis effect during the gate-sweep measurement. The degree of hysteresis is known to become higher with increasing NO_2 concentration [1]. An example of the hysteresis of gate-dependent C_{total} at 50 ppm NO_2 is shown in Figure S2c. The different C_{total} values at V_{BG} = 0 V for the sweeping directions explain the discrepancy between the response and gate-dependent measurement. The hysteresis can be reduced by increasing sweeping rate [1,2], and thus, gate voltage-dependent C_{total} can explain more precisely the capacitive NO_2 response result.

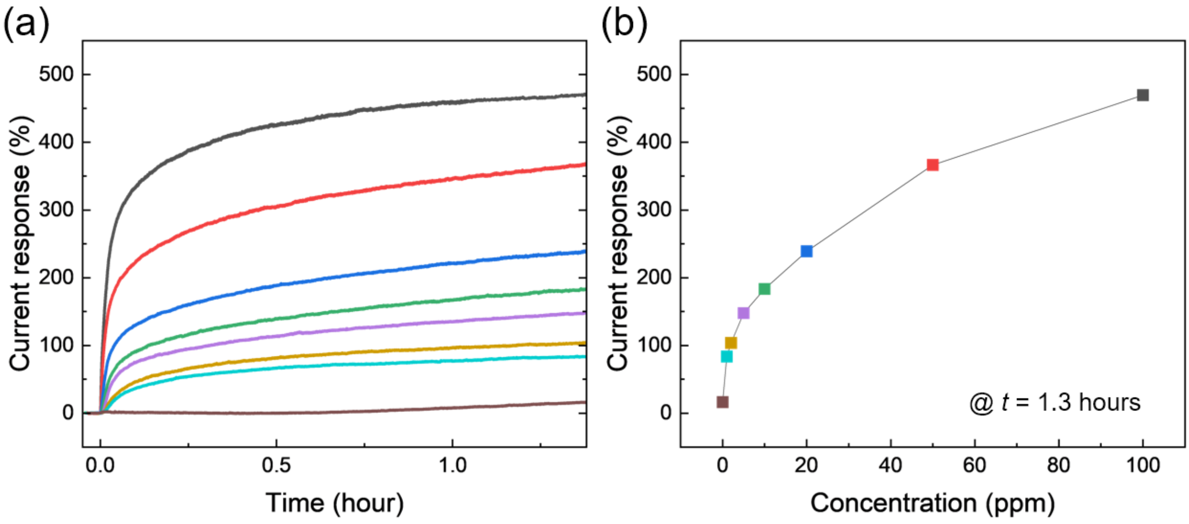

Figure S3. (**a**) The drain current response of the device using the same setup described in the main article except for the type of measurement and measuring time. The drain current of the device starts to increase when the test chamber is filled with the target concentrations of NO_2. The resistance of the device is changed by the concentrations of NO_2. The current response also depends on the concentrations of NO_2 similar to the capacitive response as shown in (**b**).

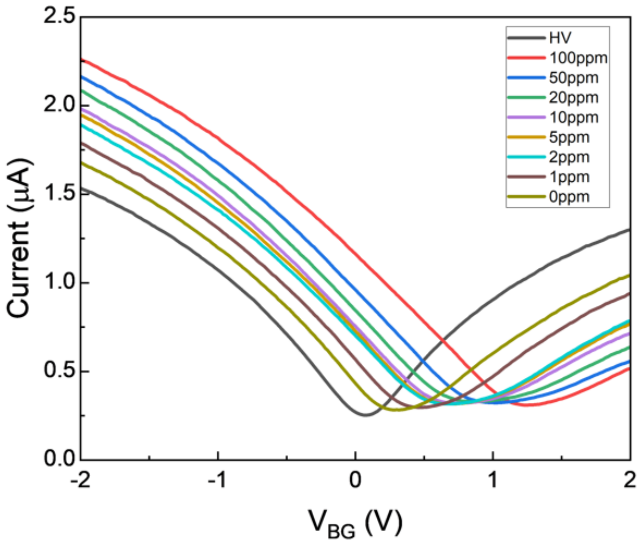

Figure S4. Gate voltage-dependent drain current of the device, which is exposed to various concentrations of NO_2, was measured after finishing the drain current response measurement. The curves are shifted to the right in accordance with the concentrations, supporting the mechanism of drain current response similar to the capacitive response.

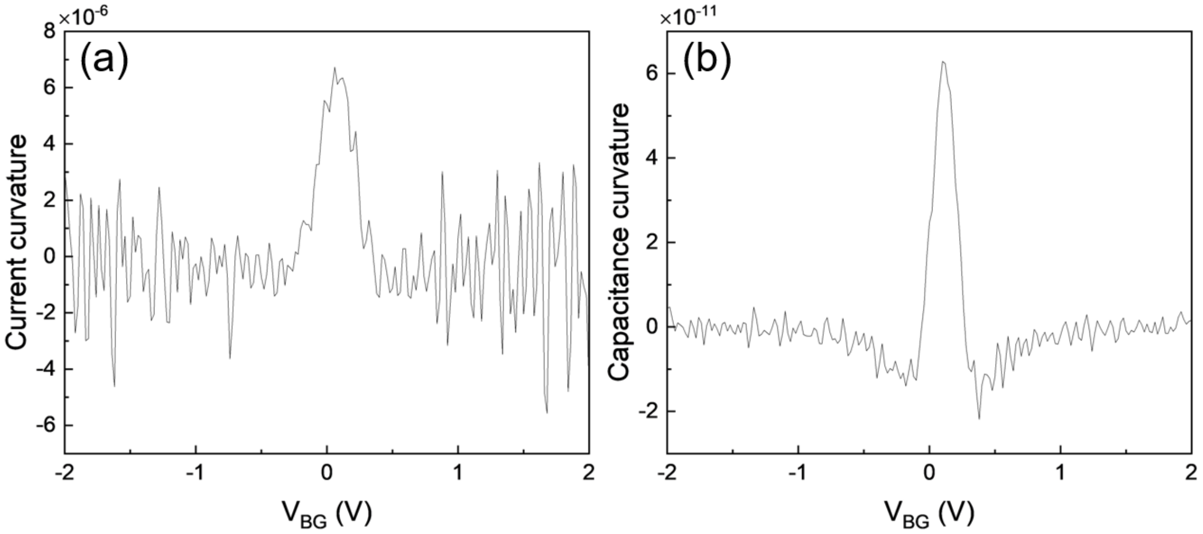

Figure S5. Curvatures are calculated using equation (S1) from the gate-dependent current in Figure 4 and Figure 6b measured under vacuum:

$$k = y''/(1 + y'^2)^{3/2} \quad \text{(S1)}$$

where k is the curvature of a curve. The curvature is the maximum value at the Dirac point and is decreased as the gate voltage increase. Full width at half maximum (FWHM) of the capacitance and current are 0.028 and 0.21, respectively. This indicates that the gate-dependent capacitance exhibits a more dramatic change in its value near Dirac point compared to the gate-dependent current measurement, indicating a sharper initial change. In turn, this proves that the capacitive measurement shows the greater sensitivity when NO_2 is introduced than the resistive measurement.

References

1. Wei, J.; Liang, B.; Cao, Q.; Ren, H.; Zheng, Y.; Ye, X. Understanding asymmetric transfer characteristics and hysteresis behaviors in graphene devices under different chemical atmospheres. *Carbon* **2020**, *156*, 67–76.
2. Wang, H.; Wu, Y.; Cong, C.; Shang, J.; Yu, T. Hysteresis of electronic transport in graphene transistors. *ACS nano* **2010**, *4*, 7221–7228.
